# Announcement: Industrial Toxicology. 99

**DOI:** 10.1155/MBD.1998.261a

**Published:** 1998


					INDUSTRIAL TOXICOLOGY 99
June 16th 18th, 1999,
Bratislava, Slovak Republic
The toxicological Chemistry Section of the Slovak Chemical Society of the Slovak Academy of
Sciences in Bratislava in cooperation with the Toxicological Section of the Slovak Society for
Industrial Chemistry organize an International Symposium
"INDUSTRIAL TOXICOLOGY.99"
June 16th 18th, 1999, Bratislava, Slovak Republic
The topics covered by the Symposium will be:
1. Chemicals and living organisms
2. Analysis and monitoring by toxicicology agencies in the environment
3. Risk analysis assessment, management of chemical technologies and use
of chemicals
4. Protection prophylaxis and treatment of intoxication
5. Miscellaneous
For details write to'
Prof. Ag,ta Fargasov,, Ph.D.
Department of Environmental Science
Faculty of Chemical Technology
SIovak University of Technology
Radlinskho 9, SK-812 37 Bratislava
SIovak Republic
Tel.: +421-7-5325 232
Fax: +42-7-393198
E-mail: bodik@chelin.chtf.stuba.sk
261

				

